# Analysis of fall-related adverse events among older adults using the Japanese Adverse Drug Event Report (JADER) database

**DOI:** 10.1186/s40780-018-0129-8

**Published:** 2018-12-17

**Authors:** Haruna Hatahira, Shiori Hasegawa, Sayaka Sasaoka, Yamato Kato, Junko Abe, Yumi Motooka, Akiho Fukuda, Misa Naganuma, Satoshi Nakao, Ririka Mukai, Kazuyo Shimada, Kouseki Hirade, Takeshi Kato, Mitsuhiro Nakamura

**Affiliations:** 1 Laboratory of Drug Informatics, Gifu Pharmaceutical University, 1-25-4, Daigaku nishi, Gifu, 501-1196 Japan; 2 Medical Database Co., Ltd., 3-11-10 Higashi, Shibuya-ku, Tokyo, 150-0011 Japan; 3 Department of Pharmacy, Kizawa Memorial Hospital, Kobi-cho, Shimo-kobi 590, Minokamo-shi, Gifu, 505-8503 Japan

**Keywords:** Fall, Benzodiazepine, Hypnotics and sedatives, Calcium channel blockers, Adverse event reporting system, JADER

## Abstract

**Background:**

Falls are a common but serious problem in older adults, and may lead to fractures and bleeding. As many factors, such as medication, aging, and comorbid diseases may simultaneously affect fall-related adverse events (AEs) in older adults, we evaluated the association between fall-related AEs and the use of medication, aging, and comorbid diseases using the Japanese Adverse Drug Event Report (JADER) database.

**Methods:**

We analyzed reports of fall-related AEs associated with α-blockers, diuretics, calcium channel blockers, central nervous system (CNS)-active drugs (opioids, benzodiazepines, hypnotics and sedatives, non-selective monoamine reuptake inhibitors, and selective serotonin reuptake inhibitors (SSRI)) in the JADER database using the reporting odds ratio (ROR). For the definition of falls, we used the Preferred Terms of The Medical Dictionary for Regulatory Activities (MedDRA). We used the association rule mining technique to discover undetected associations, such as potential risk factors.

**Results:**

The JADER database comprised 430,587 reports between April 2004 and November 2016. The RORs (95% CI) of α-blockers, diuretics, calcium channel blockers, opioids, benzodiazepines, hypnotics and sedatives, non-selective monoamine reuptake inhibitors, and SSRIs were 1.63 (1.27–2.09), 0.74 (0.63–0.86), 1.26 (1.15–1.38), 0.93 (0.80–1.07), 1.83 (1.68–2.01), 1.55 (1.12–2.14), 2.31 (1.82–2.95), and 2.86 (2.49–3.29), respectively. From the *lift* value in the association rule mining, the number of administered CNS-active drugs and patient age were associated with fall-related AEs. Furthermore, the scores of *lift* for patients with herpes zoster administered calcium channel blockers or benzodiazepines and patients with dementia administered benzodiazepines were high.

**Conclusion:**

Our results suggest that the number of administered CNS-active drugs and patient age are both associated with fall-related AEs. We recommend that patients with herpes zoster treated with calcium channel blockers and benzodiazepines be closely monitored for fall-related AEs.

## Background

Falls are common health events and a serious problem among older adults [1–3]. Falls may cause severe fractures, functional decline, decreased activity, and reduction in quality of life [4–7]. Risk factors for falls among older adults include intrinsic, extrinsic, and environmental factors [8–10]. Intrinsic factors are age-related changes in the sensory motor system leading to gait/balance disorder, dysfunctions of the nervous and muscular systems, dizziness/vertigo, postural hypotension, and visual disorders. Extrinsic factors include medications [9, 11–14].

Several medication classes, including antidepressants, antipsychotics, benzodiazepines, sedative hypnotics, opioids, some cardiac drugs, and diuretics, have been associated with an increased risk of falls [8–15]. The Japan Geriatric Society published guidelines for safe pharmacotherapy in the elderly. Potentially inappropriate medication uses were summarized in the guideline as follows: benzodiazepines, non-benzodiazepines, and anxiolytics associated with falls and related fractures; antidepressants (tricyclic antidepressants) associated with orthostatic hypotension; loop diuretics and α-blockers associated with orthostatic hypotension and falls [16]. The use of benzodiazepines and sedative hypnotics can increase the risk of falls [9, 17–19] due to dizziness, sedation, impaired motor coordination, and postural disturbances [20]. Medications affecting the central nervous system (CNS) can cause dizziness and orthostatic hypotension that increases the risk of falls [8, 9, 21, 22].

According to the American Geriatrics Society (AGS) 2015 Updated Beers Criteria, taking 3 or more CNS-active drugs concomitantly increases the risk of falls [15]. On the other hand, polypharmacy can lead to drug interactions and may be an important risk factor for falls [23]. Previous studies have reported an association between polypharmacy and falls [8, 24–29], although some studies found no association [25, 30–32]. To our knowledge, the detailed association between the number of concomitant CNS-active drugs and falls remains unclear.

The spontaneous reporting system (SRS), such as the US Food and Drug Administration (FDA) adverse event reporting system (FAERS), has been used in pharmacovigilance assessments [33, 34]. Based on the FAERS database, we previously reported that the concomitant use of antipsychotic drugs may increase the risk of hyperglycemic adverse events (AEs) using established pharmacovigilance indexes of the reporting odds ratio (ROR) [35]. The regulatory authority in Japan, the Pharmaceuticals and Medical Devices Agency (PMDA), controls the SRS of the Japanese Adverse Drug Event Report (JADER) database. By assessing the adjusted RORs using the JADER database, we demonstrated that polypharmacy may be more closely associated with an increased risk of renal disorder than hepatic disorder [36].

Association rule mining has been proposed as a new analytical approach for identifying undetected associations between variables in large databases, such as potential risk factors. Recently, this algorithm has been applied to assess the association rules of AEs in the JADER database [37–40]. As many factors, such as medications, aging, and comorbid diseases, may be simultaneously affecting fall-related AEs in older adults [41–44], we included these factors in our analysis.

In the present study, we aimed to explore the association between fall-related AEs and the use of medications such as antidepressants, antipsychotics, benzodiazepines, sedative hypnotics, opioids, calcium channel blockers, and diuretics with ROR using the SRS database. To the best of our knowledge, this is the first study to evaluate potential associations among the number of concomitant CNS-active drugs, aging, and fall-related AEs using the association rule mining technique. Furthermore, we explore the association rules among fall-related AEs, the use of medication, and comorbid diseases.

## Methods

### Data sources

Data from the JADER database between April 2004 and November 2016 were downloaded from the PMDA website (www.pmda.go.jp). The database consists of 4 data tables: patient demographic information (DEMO), drug information (DRUG), AEs (REAC), and primary disease (HIST). We constructed a relational database integrating the 4 tables using FileMaker Pro Advanced 13 (FileMaker, Inc. Santa Clara, CA). The description of age was recorded in the data table of DEMO that includes patient demographic information. The reports were stratified by age as follows: ≤19, 20–29, 30–39, 40–49, 50–59, 60–69, 70–79, 80–89, and ≥ 90 years. If the description of age was included as young adults, adults, elderly, first trimester, second trimester, third trimester, or unknown, the patient was excluded because these descriptions could not be categorized into precise 10-year intervals.

### Drugs

We used the Anatomical Therapeutic Chemical (ATC) Classification System described by the World Health Organization (WHO) Collaborating Centre for Drug Statistics Methodology for drug definition. All generic names of drugs were verified and subsequently linked with the corresponding ATC classification code. According to the listed drugs in the AGS 2015 Updated Beers Criteria, 98 drugs were selected and categorized into 8 ATC-drug classes: α-blockers (“α-adrenoreceptor antagonists” (ATC code: C02CA, https://www.whocc.no/atc_ddd_index/?showdescription=yes&code=C02CA)); diuretics (“sulfonamides, plain” (ATC code: C03CA, https://www.whocc.no/atc_ddd_index/?code=C03CA), “aryloxyacetic acid derivatives” ATC code: C03CC, https://www.whocc.no/atc_ddd_index/?showdescription=yes&code=C03CC)); calcium channel blockers (“calcium channel blockers” (ATC code: C08, https://www.whocc.no/atc_ddd_index/?code=C08)); opioids (“opioids” (ATC code: N02A, https://www.whocc.no/atc_ddd_index/?code=N02A)); benzodiazepines (“benzodiazepine derivatives” (ATC code: N05CD, https://www.whocc.no/atc_ddd_index/?code=N05CD), “benzodiazepine related drugs” (ATC code: N05CF, https://www.whocc.no/atc_ddd_index/?code=N05CF)); hypnotics and sedatives (“barbiturates, plain” (ATC code: N05CA, https://www.whocc.no/atc_ddd_index/?code=N05CA), “aldehydes and derivatives” (ATC code: N05CC, https://www.whocc.no/atc_ddd_index/?code=N05CC), “melatonin receptor agonists” (ATC code: N05CH, https://www.whocc.no/atc_ddd_index/?code=N05CH), “other hypnotics and sedatives” (ATC code: N05CM, https://www.whocc.no/atc_ddd_index/?code=N05CM)); non-selective monoamine reuptake inhibitors (“non-selective monoamine reuptake inhibitors” (ATC code: N06AA, https://www.whocc.no/atc_ddd_index/?code=N06AA)); SSRI (“selective serotonin reuptake inhibitors” (ATC code: N06AB, https://www.whocc.no/atc_ddd_index/?showdescription=yes&code=N06AB)) (Table 1).

**Table 1:** Suspected drugs classified by the Anatomical Therapeutic Chemical classification system and the Defined Daily Dose (ATC/DDD)

ATC/DDD	Compounds (code no.)
*Alpha-adrenoreceptor antagonists*	prazosin (C02CA01), doxazosin (C02CA04), urapidil (C02CA06)
*Sulfonamides, plain/Aryloxyacetic acid derivatives*	frosemide (C03CA01), bumetanide (C03CA02), piretanide (C03CA03), torasemide (C03CA04), ethacrynic acid (C03CC01)
*Calcium channel blockers*	amlodipine (C08CA01), felodipine (C08CA02), nicardipine (C08CA04), nifedipine (C08CA05), nisoldipine (C08CA07), nitrendipine (C08CA08), nilvadipine (C08CA10), manidipine (C08CA11), barnidipine (C08CA12), cilnidipine (C08CA14), benidipine (C08CA15), verapamil (C08DA01), diltiazem (C08DB01), bepridil (C08EA02)
*Opioids*	morphine (N02AA01), opium (N02AA02), oxycodone (N02AA05), dihydrocodeine (N02AA08), morphine, combinations (N02AA51), hydromorphone and naloxone (N02AA53), oxycodone and naloxone (N02AA55), oxycodone and naltrexone (N02AA56), dihydrocodeine, combinations (N02AA58), codeine, combinations excl. psycholeptics (N02AA59), codeine, combinations with psycholeptics (N02AA79), pethidine (N02AB02), fentanyl (N02AB03), pethidine, combinations excl. psycholeptics (N02AB52), pethidine, combinations with psycholeptics (N02AB72), methadone, combinations excl. psycholeptics (N02AC52), dextropropoxyphene, combinations excl. psycholeptics (N02AC54), dextropropoxyphene, combinations with psycholeptics (N02AC74), pentazocine (N02AD01), buprenorphine (N02AE01), butorphanol (N02AF01), morphine and antispasmodics (N02AG01), pethidine and antispasmodics (N02AG03), dihydrocodeine and paracetamol (N02AJ01), dihydrocodeine and acetylsalicylic acid (N02AJ02), dihydrocodeine and other non-opioid analgesics (N02AJ03), codeine and paracetamol (N02AJ06), codeine and acetylsalicylic acid (N02AJ07), codeine and ibuprofen (N02AJ08), codeine and other non-opioid analgesics (N02AJ09), tramadol and paracetamol (N02AJ13), tramadol and dexketoprofen (N02AJ14), tramadol and other non-opioid analgesics (N02AJ15), oxycodone and paracetamol (N02AJ17), oxycodone and acetylsalicylic acid (N02AJ18), oxycodone and ibuprofen (N02AJ19), tramadol (N02AX02), tapentadol (N02AX06)
*Benzodiazepine derivatives/Benzodiazepine related drugs*	flurazepam (N05CD01), nitrazepam (N05CD02), flunitrazepam (N05CD03), estazolam (N05CD04), triazoram (N05CD05), lormetazepam (N05CD06), midazolam (N05CD08), brotizolam (N05CD09), quazepam (N05CD10), zopiclone (N05CF01), zolpidem (N05CF02), eszopiclone (N05CF04)
*Barbiturates, plain, Aldehydes, Melatonin receptor agonists, and other hypnotics and sedatives*	pentobarbital (N05CA01), amobarbital (N05CA02), barbital (N05CA04), secobarbital (N05CA06), thiopental (N05CA19), chloral hydrate (N05CC01), melatonin (N05CH01), ramelteon (N05CH02), bromisoval (N05CM03), scopolamine (N05CM05), triclofos (N05CM07), apronal (N05CM12), dexmedetomidine (N05CM18)
*Non-selective monoamine reuptake inhibitors*	desipramine (N06AA01), imipramine (N06AA02), clomipramine (N06AA04), trimipramine (N06AA06), lefepramine (N06AA07), amitriptyline (N06AA09), nortriptyline (N06AA10), dosulepin (N06AA16), amoxapine (N06AA17)
*Selective serotonin reuptake inhibitors*	paroxetine (N06AB05), sertraline (N06AB06), fluvoxamine (N06AB08), escitalopram (N06AB10)

CNS-active drugs were defined in the AGS 2015 Updated Beers Criteria [15]. According to the AGS criteria, we defined CNS-active drugs by combining opioids (ATC code: N02AA, N02AB, N02AC, N02AD, N02AE, N02AF, N02AG, and N02AJ), benzodiazepines (ATC code: N05CD and N05CF), hypnotics and sedatives (ATC code: N05CA, N05CC, N05CH, and N05CM), non-selective monoamine reuptake inhibitors (ATC code: N06AA) and SSRI (ATC code: N06AB) for the association analysis of the number of concomitant CNS-active drugs. For the association analysis of the number of calcium channel blockers, we defined calcium channel blockers (ATC code: C08CA, C08DA, C08DB, and C08EA). In the DRUG table, the causality of each drug was assigned a code according to its association with the AEs, such as a “suspected drug,” “concomitant drug,” or “interacting drug.” Reports with the drug code “suspected drug,” “concomitant drug,” and “interacting drug” were included in this analysis.

### Definition of adverse event

AEs in the JADER database are coded with terms in the Medical Dictionary for Regulatory Activities (MedDRA), which is the terminology dictionary used in the JADER database (the International Council for Harmonisation of Technical Requirements for Pharmaceuticals for Human Use (ICH), Introductory Guide MedDRA Version 19.0). We extracted reports of fall-related AEs using the following preferred terms (PT): fall (PT code: 10016173), dizziness (PT code: 10013573), and orthostatic hypotension (PT code: 10031127).

### Reporting odds ratio

**Fig. 1:**
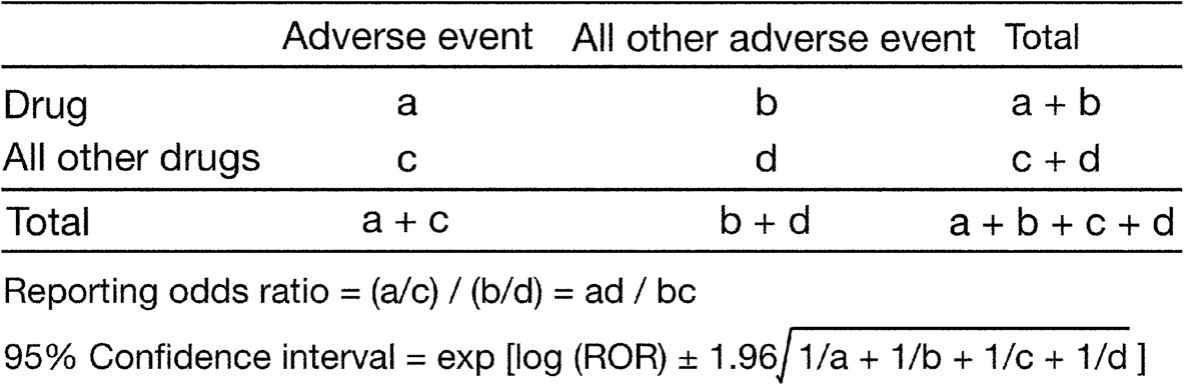
Two-by-two contingency table for the calculation of the reporting odds ratio

To detect fall-related AEs, we calculated the ROR, which is widely used in post-marketing studies, by using the disproportionality analysis. The ROR is the ratio of odds of reporting an AE (fall-related AEs) versus all other events associated with the given drug compared with the reporting odds for all other drugs in the JADER database. To compare 1 of the index groups with the reference group, we calculated the crude ROR as *(a/c)/(b/d)* (Fig. 1) [33]. ROR was expressed as point estimates with a 95% confidence interval (CI). Safety signals were considered significant when the lower limit of the 95% CI of the estimated ROR was greater than 1. Two or more cases were required to define the signal.

### Association rule mining

Association rule mining focuses on finding frequent co-occurring associations among a collection of items. Given a set of transactions T (each transaction is a set of items), an association rule can be expressed as X [the antecedent (left-hand-side, lhs) of rule:] → Y [the consequent (right-hand-side, rhs) of rule:], where X and Y are mutually exclusive sets of items. *Support* is defined as the percentage of transactions in the data that contain all items in both the antecedent (lhs) and the consequent (rhs) of the rule. The *support* indicates how frequently the rule occurs in the transaction. The formula for calculating *support* is as follows:

$$ Support=P\ \left(X\cap Y\right)=\left\{X\cap Y\right\}/\left\{D\right\} $$

D is the total number of the transaction.

*Confidence* corresponds to the conditional probability P (Y|X). *Confidence* measures the reliability of the interference made by a rule. The formula for calculating *confidence* is as follows:

$$ Confidence=P\ \left(X\cap Y\right)/P\ (X) $$

The *lift* of an association rule is frequently used to gauge the interestingness of a rule and represents the ratio of probability. The *lift* is the *confidence* divided by the proportion of all cases that are covered by the rhs. In other words, *lift* is the ratio between the *confidence* of the rule and the *support* of the itemset in the consequent of the rule. The formula for calculating *lift* is as follows:

$$ Lift=P\ \left(X\cap Y\right)/P\ (X)\ P\ (Y) $$

Lift is a measure of the importance of the association and it is independent of coverage. Since P (Y) appears in the denominator of the *lift* measure, the *lift* can be expressed as the confidence divided by P (Y). The *lift* can be evaluated as follows: *lift* = 1, if X and Y are independent; *lift* > 1, if X and Y are positively correlated; *lift* < 1, if X and Y are negatively correlated. The statistical significance of the association rule can be estimated by using the Chi-squared analysis [45, 46]. The Chi-squared statistic is defined in terms of the *confidence*, *support*, and *lift* of the single rule. We calculated the Chi-squared values to evaluate the association rules:

$$ Chi- squared=D{\left( lift-1\right)}^2\frac{Support\ast Confidence}{\left( Confidence- Support\right)\ast \left( Lift- Confidence\right)} $$

The association rule mining was performed using the *apriori* function of the *arules* package of R version 3.3.3 software. The first step of apriori algorithm searches for item sets that have more than a given minimum *support*, while in the second step, rules are generated by selecting “*confident*” item sets from those found in the first step. *Support* and *lift* were visualized using the R-extention package *arulesViz* which implements novel visualization techniques to explore association rules. The arguments of plot in the *arulesViz* were set as follows: method = “graph,” measure = “*support*,” shading = “*lift*.” The measures of *support* were used in visualization as area of circle. The measures of *lift* were used for the shading of color of the circle.

## Results

The JADER database contained 430,587 reports between April 2004 and November 2016. The number of reports for the ≥60 year-old-group was 247,170. The number of reports including fall-related AEs was 3715 overall and 2340 in the ≥60 year-old-group. The RORs (95% CI) of α-blockers, diuretics, calcium channel blockers, opioids, benzodiazepines, hypnotics and sedatives, non-selective monoamine reuptake inhibitors, and SSRI were 1.63 (1.27–2.09), 0.74 (0.63–0.86), 1.26 (1.15–1.38), 0.93 (0.80–1.07), 1.83 (1.68–2.01), 1.55 (1.12–2.14), 2.31 (1.82–2.95), and 2.86 (2.49–3.29), respectively (Table 2).

**Table 2:** Reporting odds ratio of fall-related adverse events

Drug	Case	Total	Reporting Ratio (%)	ROR (95% CI)
Total	3715	430,587		
Alpha-adrenoreceptor antagonists	64	4600	1.39	1.63 (1.27–2.09)
Sulfonamides, plain/Aryloxyacetic acid derivatives	162	25,016	0.65	0.74 (0.63–0.86)
Calcium channel blockers	542	51,482	1.05	1.26 (1.15–1.38)
Opioids	189	23,530	0.80	0.93 (0.80–1.07)
Benzodiazepine derivatives/Benzodiazepine related drugs	561	38,300	1.46	1.83 (1.68–2.01)
Barbiturates, plain, Aldehydes, Melatonin receptor agonists, and other hypnotics and sedatives	38	2865	1.33	1.55 (1.12–2.14)
Non-selective monoamine reuptake inhibitors	68	3479	1.95	2.31 (1.82–2.95)
Selective serotonin reuptake inhibitors	212	9058	2.34	2.86 (2.49–3.29)

**Fig. 2:**
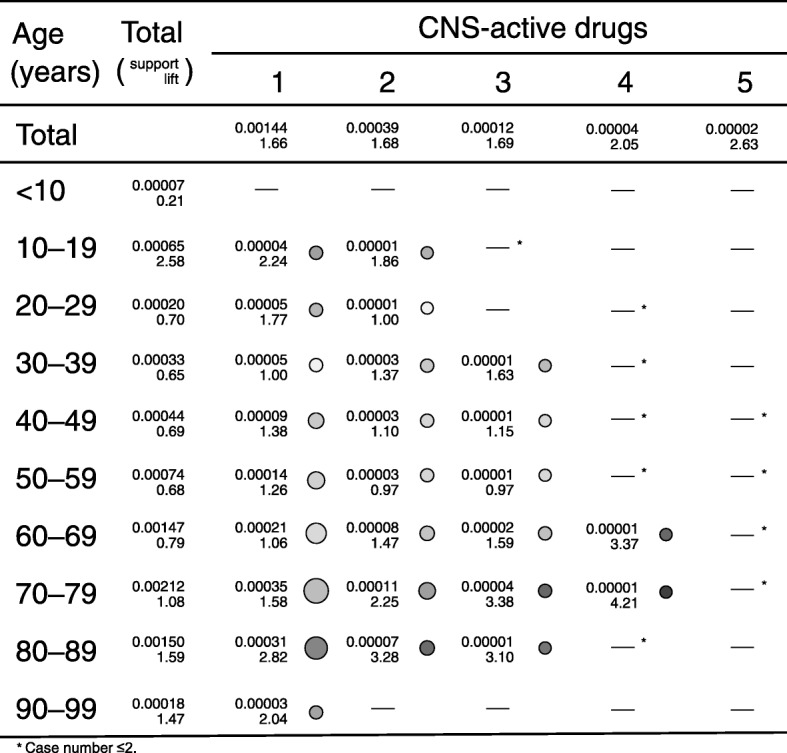
Association rules for falls-related adverse events based on JADER database between April 2004 and November 2016. *Support* and *lift* were visualized using the R-extention package *arulesViz* which implements novel visualization techniques to explore association rules. The arguments of plot in the *arulesViz* were set as follows: method = “graph,” measure = “*support*,” shading = “*lift*.” The measures of *support* were used in visualization as area of circle. The measures of *lift* were used for the shading of color of the circle

The association rule mining technique was applied to fall-related AEs (as consequent) using demographic data, such as the age category and the number of CNS-active drugs or calcium channel blockers administered (Table 3). To extract association rules efficiently, the thresholds for the optimized *support* and *confidence* thresholds were set at 0.000001 and 0.001, respectively, and *maxlen* (a parameter in the *arules* package) was restricted to 3. The number of extracted rules was 58 (Table 3). We visualized the result in the heat map of the *lift* and *support* obtained from the number of administered drugs (CNS-active drugs) and the stratified age group (Table 3, Fig. 2).

**Table 3:** Association parameters of rules based on the number of administered drugs and the stratified age group (sort by lift)

id	lhs		rhs	case (*n*)	support	confidence	lift	Chi-squared value
[1]	{4 CNS-active drugs, 70–79 years}	=>	{fall-related events}	4	0.00001	0.0364	4.21	9.90^a^
[2]	{1 calcium channel blocker, 4 CNS-active drugs}	=>	{fall-related events}	4	0.00001	0.0333	3.86	8.57^a^
[3]	{2 calcium channel blockers, 90–99 years}	=>	{fall-related events}	3	0.00001	0.0326	3.78	6.19^a^
[4]	{3 CNS-active drugs, 70–79 years}	=>	{fall-related events}	16	0.00004	0.0291	3.38	27.05^a^
[5]	{4 CNS-active drugs, 60–69 years}	=>	{fall-related events}	5	0.00001	0.0291	3.37	8.41^a^
[6]	{2 CNS-active drugs, 80–89 years}	=>	{fall-related events}	28	0.00007	0.0283	3.28	44.99^a^
[7]	{3 CNS-active drugs, 80–89 years}	=>	{fall-related events}	5	0.00001	0.0267	3.10	7.17^a^
[8]	{1 CNS-active drug, 80–89 years}	=>	{fall-related events}	134	0.00031	0.0243	2.82	160.67^a^
[9]	{5 CNS-active drugs}	=>	{fall-related events}	7	0.00002	0.0227	2.63	7.16^a^
[10]	{10–19 years}	=>	{fall-related events}	280	0.00065	0.0222	2.58	280.55^a^
[11]	{2 CNS-active drugs, 70–79 years}	=>	{fall-related events}	49	0.00011	0.0194	2.25	34.46^a^
[12]	{1 CNS-active drug, 10–19 years}	=>	{fall-related events}	17	0.00004	0.0193	2.24	11.75^a^
[13]	{1 CNS-active drug, 2 calcium channel blockers}	=>	{fall-related events}	11	0.00003	0.0179	2.08	6.20^a^
[14]	{4 CNS-active drugs}	=>	{fall-related events}	19	0.00004	0.0177	2.05	10.31^a^
[15]	{1 CNS-active drug, 90–99 years}	=>	{fall-related events}	11	0.00003	0.0176	2.04	5.86^a^
[16]	{10–19 years, 2 CNS-active drugs}	=>	{fall-related events}	3	0.00001	0.0160	1.86	1.20
[17]	{1 calcium channel blocker, 80–89 years}	=>	{fall-related events}	143	0.00033	0.0153	1.77	49.22^a^
[18]	{1 CNS-active drug, 20–29 years}	=>	{fall-related events}	21	0.00005	0.0152	1.77	7.07^a^
[19]	{2 calcium channel blockers, 50–59 years}	=>	{fall-related events}	6	0.00001	0.0147	1.70	1.75
[20]	{2 calcium channel blockers, 70–79 years}	=>	{fall-related events}	18	0.00004	0.0146	1.69	5.12^a^
[21]	{3 CNS-active drugs}	=>	{fall-related events}	50	0.00012	0.0146	1.69	14.29^a^
[22]	{2 CNS-active drugs}	=>	{fall-related events}	169	0.00039	0.0145	1.68	48.38^a^
[23]	{1 calcium channel blocker, 3 CNS-active drugs}	=>	{fall-related events}	7	0.00002	0.0144	1.67	1.91
[24]	{1 CNS-active drug}	=>	{fall-related events}	622	0.00144	0.0143	1.66	183.30^a^
[25]	{3 CNS-active drugs, 30–39 years}	=>	{fall-related events}	6	0.00001	0.0141	1.63	1.47
[26]	{80–89 years}	=>	{fall-related events}	644	0.00150	0.0137	1.59	158.61^a^
[27]	{3 CNS-active drugs, 60–69 years}	=>	{fall-related events}	10	0.00002	0.0137	1.59	2.19
[28]	{1 CNS-active drug, 70–79 years}	=>	{fall-related events}	151	0.00035	0.0136	1.58	33.23^a^
[29]	{1 CNS-active drug, 1 calcium channel blocker}	=>	{fall-related events}	102	0.00024	0.0128	1.48	16.31^a^
[30]	{2 CNS-active drugs, 60–69 years}	=>	{fall-related events}	34	0.00008	0.0127	1.47	5.25^a^
[31]	{90–99 years}	=>	{fall-related events}	76	0.00018	0.0127	1.47	11.73^a^
[32]	{1 calcium channel blocker, 90–99 years}	=>	{fall-related events}	15	0.00003	0.0121	1.40	1.72
[33]	{1 CNS-active drug, 40–49 years}	=>	{fall-related events}	38	0.00009	0.0119	1.38	4.07^a^
[34]	{2 CNS-active drugs, 30–39 years}	=>	{fall-related events}	11	0.00003	0.0118	1.37	1.09
[35]	{1 calcium channel blocker, 70–79 years}	=>	{fall-related events}	182	0.00042	0.0110	1.28	11.44^a^
[36]	{1 CNS-active drug, 50–59 years}	=>	{fall-related events}	61	0.00014	0.0108	1.26	3.28
[37]	{2 calcium channel blockers}	=>	{fall-related events}	39	0.00009	0.0108	1.25	2.00
[38]	{1 calcium channel blocker, 2 CNS-active drugs}	=>	{fall-related events}	20	0.00005	0.0107	1.24	0.97
[39]	{1 calcium channel blocker}	=>	{fall-related events}	502	0.00117	0.0105	1.22	23.01^a^
[40]	{3 CNS-active drugs, 40–49 years}	=>	{fall-related events}	5	0.00001	0.0099	1.15	0.10
[41]	{2 CNS-active drugs, 40–49 years}	=>	{fall-related events}	12	0.00003	0.0095	1.10	0.12
[42]	{70–79 years}	=>	{fall-related events}	913	0.00212	0.0093	1.08	6.35^a^
[43]	{1 CNS-active drug, 60–69 years}	=>	{fall-related events}	91	0.00021	0.0091	1.06	0.30
[44]	{1 calcium channel blocker, 60–69 years}	=>	{fall-related events}	109	0.00025	0.0090	1.04	0.16
[45]	{2 CNS-active drugs, 20–29 years}	=>	{fall-related events}	5	0.00001	0.0086	1.00	0.000003^a^
[46]	{1CNS-active drug, 30–39 years}	=>	{fall-related events}	21	0.00005	0.0086	1.00	0.0005^a^
[47]	{3 CNS-active drugs, 50–59 years}	=>	{fall-related events}	5	0.00001	0.0084	0.97	0.0035^a^
[48]	{2 CNS-active drugs, 50–59 years}	=>	{fall-related events}	15	0.00003	0.0084	0.97	0.01
[49]	{1 calcium channel blocker, 40–49 years}	=>	{fall-related events}	12	0.00003	0.0077	0.89	0.17
[50]	{2 calcium channel blockers, 80–89 years}	=>	{fall-related events}	5	0.00001	0.0070	0.81	0.22
[51]	{60–69 years}	=>	{fall-related events}	634	0.00147	0.0068	0.79	44.85^a^
[52]	{20–29 years}	=>	{fall-related events}	88	0.00020	0.0060	0.70	12.29^a^
[53]	{40–49 years}	=>	{fall-related events}	190	0.00044	0.0060	0.69	28.47^a^
[54]	{50–59 years}	=>	{fall-related events}	318	0.00074	0.0059	0.68	54.71^a^
[55]	{2 calcium channel blockers, 60–69 years}	=>	{fall-related events}	5	0.00001	0.0058	0.67	0.82
[56]	{30–39 years}	=>	{fall-related events}	140	0.00033	0.0056	0.65	27.93^a^
[57]	{1 calcium channel blocker, 50–59 years}	=>	{fall-related events}	27	0.00006	0.0056	0.65	5.25^a^
[58]	{1 calcium channel blocker, 30–39 years}	=>	{fall-related events}	3	0.00001	0.0048	0.55	1.10

^a^Statistical significance: Chi-squared value ≥4

For fall-related AEs caused by CNS-active drugs, a greater number of administered CNS-active drugs showed a trend of higher *lift* because the *lift* of 1 medication, 2 medications, 3 medications, 4 medications, and 5 medications consisting of CNS-active drugs were 1.66, 1.68, 1.69, 2.05, and 2.63, respectively (Table 3, Fig. 2). The *lift* values increased according to the interaction between aging and the number of administered CNS-active drugs.

For fall-related AEs by calcium channel blockers, the *lift* of monotherapy and 2 *calcium channel blockers* were 1.22 and 1.25, respectively, and were almost equal (Table 3). There was no association between increasing the number of calcium channel blockers and fall-related AEs. However, when 1 calcium channel blocker was administered, a higher lift was found among the ≥70 year-old-group than among the < 70 year-old-group.

To evaluate risk factors of fall-related AEs in the ≥60 year-old-group (247,170 cases) using demographic data, such as patient history and administered drugs, we applied the *apriori* algorithm (minimum *support, confidence* threshold, 0.000001, 0.01, respectively) and the *maxlen* was restricted to 4. The number of extracted rules was 45 (Table 4). The association rules of the combination of {benzodiazepines, dementia} → {fall-related AEs}, {benzodiazepines, herpes zoster} → {fall-related AEs}, {calcium channel blockers, benign prostate hypertrophy} → {fall-related AEs}, {opioids, back pain} → {fall-related AEs}, {calcium channel blockers, herpes zoster} → {fall-related AEs}, {opioids, SSRI} → {fall-related AEs} demonstrated high *lift* scores (Table 4, id [1–6]).

**Table 4:** Association parameters of rules based on the patient history and the administered drugs (sort by lift)

id	lhs		rhs	Case (*n*)	support	confidence	lift	Chi-squared value
[1]	{benzodiazepines, dementia}	=>	{fall-related AEs}	22	0.00005	0.1222	14.17	155.94^a^
[2]	{benzodiazepines, herpes zoster}	=>	{fall-related AEs}	15	0.00003	0.0806	9.35	64.77^a^
[3]	{calcium channel blockers, benign prostate hypertrophy}	=>	{fall-related AEs}	11	0.00003	0.0769	8.92	44.78^a^
[4]	{opioids, back pain}	=>	{fall-related AEs}	13	0.00003	0.0681	7.89	45.30^a^
[5]	{calcium channel blockers, herpes zoster}	=>	{fall-related AEs}	30	0.00007	0.0551	6.39	79.11^a^
[6]	{opioids, SSRI}	=>	{fall-related AEs}	18	0.00004	0.0429	4.97	33.06^a^
[7]	{benzodiazepines, insomnia}	=>	{fall-related AEs}	22	0.00005	0.0389	4.51	34.88^a^
[8]	{calcium channel blockers, dementia Alzheimer’s type}	=>	{fall-related AEs}	17	0.00004	0.0383	4.44	26.24^a^
[9]	{dementia Alzheimer’s type, benzodiazepines}	=>	{fall-related AEs}	10	0.00002	0.0361	4.18	14.04^a^
[10]	{SSRI, depression, benzodiazepines}	=>	{fall-related AEs}	30	0.00007	0.0309	3.58	32.33^a^
[11]	{calcium channel blockers, SSRI, benzodiazepines}	=>	{fall-related AEs}	15	0.00003	0.0306	3.55	15.91^a^
[12]	{type 2 diabetis mellitus, benzodiazepines}	=>	{fall-related AEs}	12	0.00003	0.0299	3.47	12.22^a^
[13]	{SSRI, depression}	=>	{fall-related AEs}	60	0.00014	0.0298	3.46	60.93^a^
[14]	{SSRI, non-selective monoamine reuptake inhibitors}	=>	{fall-related AEs}	22	0.00005	0.0282	3.26	20.05^a^
[15]	{benzodiazepines, hypertension}	=>	{fall-related AEs}	33	0.00008	0.0276	3.20	29.04^a^
[16]	{opioids, pain}	=>	{fall-related AEs}	17	0.00004	0.0266	3.08	13.84^a^
[17]	{SSRI, benzodiazepines, non-selective monoamine reuptake inhibitors}	=>	{fall-related AEs}	10	0.00002	0.0242	2.80	6.70^a^
[18]	{depression, benzodiazepines}	=>	{fall-related AEs}	39	0.00009	0.0239	2.77	25.70^a^
[19]	{SSRI}	=>	{fall-related AEs}	212	0.00049	0.0234	2.71	135.59^a^
[20]	{calcium channel blockers, benzodiazepines, α-blockers}	=>	{fall-related AEs}	12	0.00003	0.0227	2.63	7.02^a^
[21]	{calcium channel blockers, SSRI}	=>	{fall-related AEs}	24	0.00006	0.0211	2.44	11.87^a^
[22]	{SSRI, benzodiazepines}	=>	{fall-related AEs}	81	0.00019	0.0210	2.43	40.01^a^
[23]	{calcium channel blockers, osteoporosis}	=>	{fall-related AEs}	15	0.00003	0.0208	2.41	7.21^a^
[24]	{non-selective monoamine reuptake inhibitors}	=>	{fall-related AEs}	68	0.00016	0.0195	2.27	28.06^a^
[25]	{hypertension, α-blockers}	=>	{fall-related AEs}	11	0.00003	0.0191	2.21	4.22^a^
[26]	{benzodiazepines, diabetis mellitus}	=>	{fall-related AEs}	11	0.00003	0.0183	2.12	3.76^a^
[27]	{benzodiazepines, α-blockers}	=>	{fall-related AEs}	14	0.00003	0.0175	2.02	4.20^a^
[28]	{benzodiazepines, hypnotics and sedatives}	=>	{fall-related AEs}	20	0.00005	0.0166	1.92	5.11^a^
[29]	{calcium channel blockers, benzodiazepines, hypertension}	=>	{fall-related AEs}	12	0.00003	0.0165	1.91	3.02
[30]	{calcium channel blockers, hypertension}	=>	{fall-related AEs}	93	0.00022	0.0164	1.90	23.25^a^
[31]	{benzodiazepines, schizophrenia}	=>	{fall-related AEs}	41	0.00010	0.0161	1.87	9.59^a^
[32]	{calcium channel blockers, chronic hepatitis C}	=>	{fall-related AEs}	16	0.00004	0.0160	1.85	3.63
[33]	{benzodiazepines}	=>	{fall-related AEs}	561	0.00130	0.0146	1.70	102.24^a^
[34]	{calcium channel blockers, benzodiazepine}	=>	{fall-related AEs}	122	0.00028	0.0146	1.69	20.16^a^
[35]	{α-blockers}	=>	{fall-related AEs}	64	0.00015	0.0139	1.61	8.72^a^
[36]	{type 2 diabetis mellitus, calcium channel blockers}	=>	{fall-related AEs}	20	0.00005	0.0138	1.60	2.60
[37]	{benzodiazepines, non-selective monoamine reuptake inhibitors}	=>	{fall-related AEs}	19	0.00004	0.0137	1.59	2.39
[38]	{calcium channel blockers, α-blockers}	=>	{fall-related AEs}	43	0.00010	0.0135	1.57	5.12^a^
[39]	{hypnotics and sedatives}	=>	{fall-related AEs}	38	0.00009	0.0133	1.54	4.16^a^
[40]	{calcium channel blockers, diabetis mellitus}	=>	{fall-related AEs}	21	0.00005	0.0132	1.53	2.24
[41]	{benzodiazepines, chronic hepatitis C}	=>	{fall-related AEs}	10	0.00002	0.0132	1.53	1.05
[42]	{benzodiazepines, rheumatoid arthritis}	=>	{fall-related AEs}	10	0.00002	0.0120	1.39	0.62
[43]	{calcium channel blockers}	=>	{fall-related AEs}	542	0.00126	0.0105	1.22	14.17^a^
[44]	{diuretics, hypertension}	=>	{fall-related AEs}	11	0.00003	0.0103	1.19	0.19
[45]	{benzodiazepines, diuretics}	=>	{fall-related AEs}	41	0.00010	0.0089	1.03	0.03

^a^Statistical significance: Chi-squared value ≥4

## Discussion

Falls can cause serious injuries and are associated with considerable morbidity and mortality, particularly among older adults. The present analysis showed that α-blockers, calcium channel blockers, and CNS-active drugs had high and significant RORs of fall-related AEs. Our study further indicated that the number of administered CNS-active drugs and aging are both associated with the *lift* value of fall-related AEs. For calcium channel blockers*,* we also found an age-related increase of *lift* value. The risk of falls on initiation of antihypertensive drugs in the elderly was reported [47]. Age-related physical and physiological changes increase the incidence of falls. Aging decreases hepatic metabolism and renal drug elimination. These changes lead to higher drug exposure and an increased risk of falls among older adults. These risks should be considered carefully in clinical practice.

The effect of the concomitant use of CNS-active drugs on postural balance may be additive and the concomitant use of CNS-active drugs increases the risk of falls [48] and fractures [49, 50], which are associated with high morbidity and mortality rates [51]. The risk of falls may be attributed to potential drug interactions between antidepressants and benzodiazepines. Our findings are consistent with recent research examining concomitant CNS-active drug use and falls among older adults [52–56], which suggests that pharmacodynamic drug interactions (e.g. involving CNS medication, muscle relaxants, opioids, and SSRI) with benzodiazepines contribute to the increased risk of falls. To the best of our knowledge, there have been no previously published reports on association rule mining analyses for CNS-active drugs using the SRS database. Our results suggest that the risk of fall-related AEs with CNS-active drug monotherapy should not be underestimated. The information derived from this study using association rule mining could complement earlier reports.

Studies regarding the association between polypharmacy and falls have been conducted, however, conclusive results have not been obtained due to small sample sizes [26–28, 30, 54], selective study populations [25, 26], or study design (cross-sectional analyses). For example, the use of benzodiazepines was associated only with an increased risk of injurious falls when used with concomitant medication. However, the use of benzodiazepines was also associated with a greater number of falls irrespective of polypharmacy [57]. In a prospective study involving a community-dwelling group aged > 60 years, polypharmacy was not associated with an increased risk of falls after adjusting for co-morbidity [57]. Polypharmacy is generally defined according to the total number of concurrent medications. We investigated the association rules with the number of CNS-active drugs or the number of calcium channel blockers. After considering the causality restraints of the current analysis, further robust epidemiological studies are recommended.

Polypharmacy is associated with an increased risk of administration of potentially inappropriate medication. According to the Beers criteria [58] and the Screening Tool of Older Person’s Prescriptions (STOPP)/Screening Tool to Alert doctors to Right Treatment (START) criteria [59, 60], healthcare professionals should optimize the medication of their patients and minimize polypharmacy to reduce AEs.

Among older adults, treatment is complicated by the high frequency of comorbidity [58]. We detected the association rules of combination of {benzodiazepines, herpes zoster} → {fall-related AEs} and {calcium channel blockers, herpes zoster} → {fall-related AEs}. Several studies have reported that valacyclovir and pregabalin induce dizziness which leads to falls [27]. Healthcare professionals should pay attention to the risk of falls among patients with herpes zoster administered calcium channel blockers or benzodiazepines*.* Healthcare professionals should conduct a thorough medication review, including past patient history, age-related physical changes, drug–drug interactions, and AEs considered as risk factors for falls [61–64]. Optimized interventions to reduce the incidence of falls among older adults should be introduced, such as stopping, switching, or reducing the number of administrated drugs and adding vitamin D [65].

A number of limitations of the analysis using SRS, such as the JADER database, should be noted. The SRS is subject to over-reporting, under-reporting, missing data, exclusion of healthy individuals, lack of a denominator, and the presence of confounding factors [66]. The target drugs in our study were selective, and not comprehensive, and was not intended to diminish the clinical importance of known drug–drug interactions not listed. Despite these limitations, we obtained reasonable results that complement or corroborate those reported in the literature. Our results provide valuable insights into prescribing drugs to older adults in a real-world clinical setting.

## Conclusion

This study is the first to evaluate the correlation between fall-related AEs and the number of concomitant CNS-active drugs, aging, and comorbid diseases using ROR and association rule mining technique based on the JADER database. Despite the inherent limitations of SRS, the number of administered CNS-active drugs and patient age were both associated with the *lift* value of fall-related AEs. The present analysis demonstrates that the incidence of fall-related AEs associated with benzodiazepines and calcium channel blockers use should be closely monitored in patients with herpes zoster. We believe that the data presented in this study will help healthcare professionals to improve the care of older patients administered different medications concomitantly.

## Abbreviations

AE: Adverse Event
AGS: American Geriatrics Society
ATC: The Anatomical Therapeutic Chemical
CI: Confidence Intervals
CNS: Central Nerves System
FAERS: FDA Adverse Event Reporting System
FDA: The US Food and Drug Administration
ICH: The International Council for Harmonisation of Technical Requirements for Pharmaceuticals for Human Use
JADER: The Japanese Adverse Drug Event Report
lhs: left-hand-side
MedDRA: The Medical Dictionary for Regulatory Activities
PMDA: The Pharmaceuticals and Medical Devices Agency
PT: Preferred Term
rhs: Right-hand-side
ROR: Reporting Odds Ratio
SRS: Spontaneous Reporting System
START: Screening Tool to Alert doctors to Right Treatment
STOPP: The Screening Tool of Older Person’s Prescriptions
WHO: The World Heath Organization
